# Negative Pressure Wound Therapy for the Treatment of the Open Abdomen and Incidence of Enteral Fistulas: A Retrospective Bicentre Analysis

**DOI:** 10.1155/2013/730829

**Published:** 2013-10-28

**Authors:** Sven Richter, Stefan Dold, Johannes P. Doberauer, Peter Mai, Jochen Schuld

**Affiliations:** ^1^Department of General, Visceral and Vascular Surgery, Fürst-Stirum-Klinik, 76646 Bruchsal, Germany; ^2^Department of General, Visceral, Vascular and Pediatric Surgery, University Hospital of Saarland, 66421 Homburg, Germany; ^3^Department of General and Visceral Surgery, Barmherzige Brüder Hospital, 80639 Munich, Germany

## Abstract

*Introduction.* The open abdomen (OA) is often associated with complications. It has been hypothesized that negative pressure wound therapy (NPWT) in the treatment of OA may provoke enteral fistulas. Therefore, we analyzed patients with OA and NPWT with special regard to the occurrence of intestinal fistulas. *Methods.* The present study included all consecutive patients with OA treated with NWPT from April 2010 to August 2011 in two hospitals. Patients' demographics, indications for OA, risk factors, complications, outcome and incidence of fistulas before, during and after NPWT were recorded. *Results.* Of 81 patients with OA, 26 had pre-existing fistulas and 55 were free from a fistula at the beginning of NPWT. Nine of the 55 patients developed fistulas during (*n* = 5) or after NPWT (*n* = 4). Seventy-five patients received ABThera therapy, 6 patients other temporary abdominal closure devices. Only diverticulitis seemed to be a significant predisposing factor for fistulas. Mortality was slightly lower for patients without fistulas. *Conclusion.* The present study revealed no correlation between occurrence of fistulas before, during, and after NWPT, with diverticulitis being the only risk factor. Fistula formation during NPWT was comparable to reports from literature. Prospective studies are mandatory to clarify the impact of NPWT on fistula formation.

## 1. Introduction

The open abdomen (OA) generally represents a sequel either of trauma or of complications following visceral surgery. Its management is challenging *per se* and can be further aggravated by complications [1–3]. Of those, intestinal fistulas are the most serious because of their association with significant morbidity and mortality [4–6]. Development of enteral fistulas is estimated up to 25% during the therapy of OA [3, 7–9], which may be caused by dryness of the small or large bowel resulting from exposition to the ambient air as well as by mechanical irritation of wound dressings [10]. In addition, important predisposing factors that contribute to higher fistula rates have been identified, such as inflammatory bowel disease, pancreatitis, diverticulitis, renal insufficiency, poor nutritional state, and ischemic conditions [5, 11, 12]. When fistulas develop, considerable amounts of stool fill both the abdominal wound and abdominal cavity, thereby aggravating the dilemma and leading to a vicious circle [3, 13]. Thus, in the management of OA, the prevention of any enteral fistula is mandatory to achieve appropriate abdominal wall closure, as this in turn represents the best way to avoid further complications [14, 15]. In the treatment of OA, manifold temporary abdominal closure (TAC) methods have been described, including skin-approximation closure [16], absorbable synthetic mesh products [17], Bogota bags [18], Barker's vacuum packing technique, and negative pressure wound therapy (NPWT) [2, 3, 19]. In recent studies, higher fascial closure rates were realized using NPWT [19–25] compared to Barker's vacuum packing technique [26, 27], skin towel clips, Bogota bag, and absorbable mesh [2, 17, 28, 29]. Although NPWT is widely used [30, 31], it is still a matter of debate whether fistulas develop due to the negative pressure applied [28, 32, 33]. According to the guidelines of the National Institute for Health and Clinical Excellence (NICE) [34] data of all patients with OA treated between January 1, 2010, to June 30, 2011 (treated with NPWT or alternatively), were submitted to the NICE audit. Herein, and supported by further literature [33], concerns have been expressed that NPWT may cause intestinal fistulas. The purpose of the present study was therefore to examine the incidence of fistulas in patients with OA undergoing NPWT, occurring either during ABThera or other TAC therapy.

## 2. Methods

Data was obtained retrospectively from patients' records of 2 German hospitals (University Hospital of Saarland, Homburg, Saar; Barmherzige Brüder Hospital, Munich). All patients with OA treated by NPWT between April 2010 and August 2011 were included into the study. Because NWPT of the open abdomen was representing the standard in the therapy of the OA in both hospitals, all patients were potentially eligible for the data analysis and were therefore included. Primary data acquisition was performed by standardized questionnaires from the patients records, partially available as electronic records from the hospital information systems (SAP, St. Leon Roth) and transferred to spreadsheet software (Microsoft Excel, Redmond, USA). The whole study design was approved by the local ethical committees of both hospitals. In most patients, the ABThera OA NPWT system (KCI Europe, Amstelveen, Netherlands) was used, consisting of a visceral protective layer (polyurethane foam enveloped in a polyethylene sheet with small fenestrations), polyurethane foams (for placement on top of the visceral protective layer), and self-adhesive polyethylene drapes (for air-tight coverage of the wound). Only a small minority of patients received other TAC devices based on the surgeon's individual decision. These latter devices consisted of a sterile and perforated plastic foil for visceral protection, directly covered with standard polyurethane foam (V.A.C. GranuFoam, KCI) and sealed with self-adhesive polyethylene drapes. NPWT in all patients, including negative pressure settings of −100 mmHg to −125 mmHg [35], and regular dressing changes were performed as recommended by the manufacturer and described elsewhere [36]. Patients received standard of care, which included intensive care unit therapy (with mechanical ventilation if necessary), oral fluids, and adequate nutrition. Patients were grouped based on presence/absence of a fistula as well as occurrence of the fistula: no fistula, preexisting fistula, fistula developed during NPWT, and fistula after termination of NPWT. Patients' demographics (e.g., gender, age, height, BMI, APACHE, and SOFA scores), indications for OA, and preexisting risk factors were recorded. Complications (including fistula formation) were analyzed and adverse events as well as patients' outcome (e.g., primary/secondary fascial closure and death) were analyzed. The occurrence of any fistula during or after NPWT was defined as dependent variable. Statistical analyses were performed using the Statistical Package for Social Sciences (SPSS statistics, Version 15.0, Chicago, SPSS Inc.). All demographic variables (e.g., patient age, height, BMI, APACHE, and SOFA scores) were expressed as mean ± standard deviation. Kruskal-Wallis rank test for quantitative data was applied. Fisher's exact test or Chi-square test was used for the comparison of no fistula group to any fistula group, for OA indications, predisposing factors, outcomes, and adverse events.

## 3. Results

Data was obtained from a total of 81 consecutive patients undergoing NPWT. Statistical analysis of patient demographics revealed no significant differences (Table 1). The majority of OA was due to surgical/nontraumatic indications. Bowel perforation, anastomotic leakage, or abdominal compartment syndrome showed a significant (*P* < 0.05) relation to the occurrence of fistulas (Table 2). All patients had at least one preexisting risk factor for fistula formation with a high percentage of patients having acute renal insufficiency or malignancy (Table 3). However, the only significant predisposing factor for fistula formation was diverticulitis, being observed only in patients with fistulas (0.0% versus 17.1%; *P* < 0.01) (Table 3). 

Seventy-five patients (92.6%) received ABThera therapy, whereas in 6 patients (7.4%) other TAC devices were used. There were 55 patients with no preexisting fistulas and 26 patients with preexisting fistulas. Out of the 55 patients with no preexisting fistulas, 5 developed fistulas during NPWT. These fistulas have become clinically evident between day 3 and day 10 after the beginning of NPWT. Three of patients with fistulas during NPWT received standard ABThera therapy, whereas the other 2 patients were treated by other TAC devices. Another 4 patients with no preexisting fistula and no fistula during NPWT developed a secondary fistula between 3 and 46 days after the end of therapy. Out of those four patients, two received other TAC devices whereas the other 2 patients received ABThera therapy. The all-in-all fistula rate during both acute NPWT and short time interval after the operation was 16.4%. 5 of the fistulas (9.1%) occurred under ABThera therapy whereas 4 fistulas (7.3%) occurred when using other TAC devices. The number of days with OA was significantly lower for patients with no fistulas compared to patients who developed fistulas during NPWT (8.2 ± 10.5 days versus 24.2 ± 13.7 days; *P* < 0.05). This correlated significantly with fewer days of NPWT for patients with no fistulas compared to patients who developed fistulas during NPWT (7.7 ± 10.2 days versus 24.2 ± 13.7 days; *P* < 0.05). The majority of OA could be closed within 30 days (84.8%) in patients with no fistula. In contrast, closure rate in the same time interval for patients with fistulas was 74.3% (*P* = 0.27) (Table 4). Overall mortality was 30.9% and slightly lower for patients without fistulas compared to those with fistulas (28.6% versus 34.3%); however, this did not reveal any significance (*P* = 0.63) (Table 4). As well, no significant difference in number of adverse events between patients with fistulas and patients without fistulas could be shown (Table 5). 

## 4. Discussion

The treatment of the open abdomen represents a surgical challenge *per se* but can be further aggravated by a variety of complications, resulting in high mortality [7, 26, 31]. It is known from the literature that NPWT results in a higher rate in secondary abdominal wall closure compared to standard care. Secondary closure rate was 84.8% in patients with no fistula overall and 74.3% in patients with any fistula. These closure rates are comparable to what is already known in the NPWT of the open abdomen [14, 19, 24, 28, 31, 32].

However, fistulas may lead to a vicious cycle as the fistula itself may impede fascial closure and thereby perpetuate and aggravate the difficult situation of the OA. Although there may be a multifactorial etiology of fistula development, dessication of the bowel is regarded as a major factor for the development of fistulas [10, 37]. Therefore, one strategy to prevent fistulas is to cover all exposed bowel with omentum, avoid hyperresuscitation and the resulting bowel edema, and minimize serosal injury to the exposed bowel. Furthermore a definitive closure of the skin or fascia therefore represents the best dressing and prevents enteroatmospheric fistulas [15]. The recently introduced ABThera device was also suspicious for causing enteral fistulas [34]. It can be hypothesized that the vacuum effect may be harmful, as it may impede enteral microcirculation, and it has been proposed hitherto never to use the vacuum sponge in direct contact to bowel serosa. Within the data presented herein, we report 9 new fistulas (16.4%) during or after NPWT, which is higher than the fistula rate reported in two recently published reviews with predominantly included trauma patients [1, 31]. 55 of our patients had no preexisting fistula at the beginning of the NPWT. During or after NPWT 9 patients developed a fistula. The fistula rate of 16.4% as shown herein is comparable to the most studies including mainly patients with peritonitis and OA (14.8–21%) [28, 38–40]. Although our patients' collective was bigger than those studies included in the above mentioned reviews, we could not determine any statistical significant parameter for fistula formation—except for colon diverticulitis. This may be explained by a higher vulnerability of the intestinal. Nevertheless, our study is representing a collective of patients with secondary peritonitis as the indicating reason for the use of NPWT. 

However, some shortcomings of the present study have to be mentioned. In general, the crunch question of NPWT in the treatment of the open abdomen is, Does NPWT helps to treat fistulas or does it cause new fistulas? One may argue that the NPWT itself is causing fistulas. Most of the patients included suffered from severe peritonitis and were critically ill being evident as high APACHE and SOFA scores. To clarify a possible adverse effect of NPWT in fistula formation, bigger homogenous collectives of patients have to be evaluated prospectively. Due to the retrospective character, an adequate power and sample size calculation has not been done. In addition, the OA treatment with NPWT represents a single arm study without any control group, so there is no way to determine if the rate of fistula would have been different with another treatment. Furthermore in one of both clinics including the majority of patients, single high volume lavage with primary closure of the fascia was preferred in the treatment of secondary peritonitis as published by our group in 2009 [41]. 

One finding of our study is that more than 40% of the fistulas developing under NPWT occurred when self-made abdominal vacuum devices were used. All four patients with self-made vacuum TACs were from one hospital but at least treated by different surgeons, what is representing a confounder variable. Although the difference in new fistula incidence seems to be evident, it is not possible to draw a final conclusion as to whether other TAC devices using NPWT are an independent factor influencing fistula rate because of the small number of patients treated with homemade TAC devices. Our study surely consists of some bias, and it remains quite difficult to hypothesize about the pathophysiology of fistula development during self-made vacuum devices. Explanations may be that with self-made vacuum devices the sponge may get in contact with the bowel or that irregular perforation holes in the foil create inhomogeneous suction levels at the intestinal site.

Another reason for the development of fistulas may consist in the changing intervals of the abdominal dressing. So far, no study exists defining the optimal time point for changing the vacuum dressing. In our experience clinical routine shows that many dressings have been redone on demand, when patients show clinical worsening such as septic complications or when the consistence of the suction fluid is changing quality. According to our results longer NPWT results in a high rate of fistulas during or after the therapy. Maybe one reason to avoid fistulas is to change the NPWT system in shorter intervals with an assessment for earlier closure of the abdominal wall or at least skin closure. As well, we cannot answer the question whether the development of fistulas correlates with the extent of vacuum pressure applied, as the vacuum settings in our retrospective data collection were not standardized and thus heterogeneous but mostly over 100 mmHg of negative pressure. However, this point may not be of too great importance, as the suction measured within the abdominal cavity seems to be quite similar at any negative vacuum pressure applied [42]. In contrast to that, recent data indicates that *lower suction intensity* [35, 39] is more preferred than higher intensity as we did it in our study. Overall mortality was comparable to previous published studies [1, 7, 19, 26]. Interestingly, the occurrence of fistula did not affect overall mortality in our study. 

All in all, it has to be kept in mind that *not all* surgeons have unlimited access to industrially standardized NPWT products as it is represented by the ABThera or similar products. In addition, most of the patients suffering from open abdomen—caused by surgical complications initially—are treated in an emergency situation. In those cases there may be often no other way to achieve a temporary closure of the abdomen other than using other TAC devices because the availability of NPWT products in the treatment of the open abdomen is not the standard in all hospitals. In addition many hospitals facing economical challenges cannot afford such cost intensive therapies. 

## 5. Conclusion

The development of fistulas during NPWT is representing a challenging and common problem in the context of peritonitis in critically ill patients. When using standardized industrially NPWT products, intestinal fistulas cannot be avoided. It can be assumed that peritonitis itself and other patient related factors may contribute to fistula development. To draw a final conclusion, if NPWT products in the therapy of the open abdomen are *causing* or *preventing* fistulas, larger databases included in prospective multicentre studies are mandatory.

## Figures and Tables

**Table 1:** Patient demographics.

	Initially no fistula (*n* = 55)			Preexisting fistula (*n* = 26)	Any fistula overall (*n* = 35)	*P* value (any fistula versus no fistula)
	No fistula overall (*n* = 46)	Fistula during NPWT (*n* = 5)	Fistula after NPWT (*n* = 4)			
Male (%)	48.8	60.0	50.0	39.5	62.9	0.44
Age (years)	67.6 ± 14.8	61.2 ± 10.0	54.8 ± 14.7	61.8 ± 20.0	60.9 ± 18.2	0.22
BMI (kg/m^2^)	26.6 ± 5.3	26.9 ± 6.6	26.4 ± 2.1	25.6 ± 4.8	25.9 ± 4.8	0.83
APACHE Score	19.3 ± 4.8	17.6 ± 6.1	17.0 ± 7.0	19.5 ± 4.6	18.9 ± 5.0	0.66
SOFA Score	7.2 ± 4.4	6.8 ± 3.3	8.8 ± 4.9	6.5 ± 4.8	6.8 ± 4.5	0.66

**Table 2:** Indications for the OA.

	Initially no fistula (*n* = 55)			Preexisting fistula (*n* = 26)	Any fistula overall (*n* = 35)	*P* value (any fistula versus no fistula)
	No fistula (*n* = 46)	Fistula during NPWT (*n* = 5)	Fistula after NPWT (*n* = 4)			
Surgical/nontrauma	95.7% (44)	100.0% (5)	100.0% (4)	96.2% (25)	97.1% (34)	1.0
Traumatic	4.4% (2)	0.0% (0)	0.0% (0)	3.9% (1)	2.9% (1)	NA
Sepsis peritonitis	50.0% (23)	60.0% (3)	50.0% (2)	80.8% (21)	74.3% (26)	0.04
Bowel perforation	19.6% (9)	40.0% (2)	50.0% (2)	61.5% (16)	57.1% (20)	<0.0001
Ischemic bowel	17.4% (8)	20.0% (1)	0.0% (0)	11.5% (3)	11.4% (4)	0.54
Pancreatitis	6.5% (3)	0.0% (0)	25.0% (1)	3.9% (1)	5.7% (2)	1.0
Intra-abdominal abscess	15.2% (7)	60.0% (3)	50.0% (2)	15.4% (4)	25.7% (9)	0.27
Anastomotic dehiscence	0.0% (0)	0.0% (0)	50.0% (2)	26.9% (7)	25.7% (9)	<0.0001
Vascular	15.2% (7)	0.0% (0)	0.0% (0)	3.9% (1)	2.9% (1)	0.13
Intra-abdominal hemorrhage	23.9% (11)	0.0% (0)	25.0% (1)	11.5% (3)	11.4% (4)	0.25
Abdominal aortic aneurysm	21.7% (10)	0.0% (0)	0.0% (0)	7.7% (2)	5.7% (2)	0.06
Damage control surgery	4.4% (2)	0.0% (0)	25.0% (1)	3.9% (1)	5.7% (2)	1.0
Tumor resection	13.0% (6)	0.0% (0)	25.0% (1)	15.4% (4)	14.3% (5)	1.0
Other indication	30.4% (14)	20.0% (1)	0.0% (0)	15.4% (4)	14.3% (5)	0.12

**Table 3:** Predisposing factors.

	Initially no fistula (*n* = 55)			Preexisting fistula (*n* = 26)	Any fistula overall (*n* = 35)	*P* value (any fistula versus no fistula)
	No fistula overall (*n* = 46)	Fistula during NPWT (*n* = 5)	Fistula after NPWT (*n* = 4)			
Inflammatory bowel disease	8.7% (4)	20.0% (1)	0.0% (0)	7.7% (2)	8.6% (3)	1.0
Diverticulitis	0.0% (0)	20.0% (1)	50.0% (2)	11.5% (3)	17.1% (6)	0.01
Pancreatitis	8.7% (4)	20.0% (1)	25.0% (1)	7.7% (2)	11.4% (4)	0.72
Cholecystitis	15.2% (7)	20.0% (1)	25.0% (1)	19.2% (5)	20.0% (7)	0.77
Radiation therapy	0.0% (0)	0.0% (0)	0.0% (0)	0.0% (0)	0.0% (0)	NA
Poor nutritional status	6.5% (3)	20.0% (1)	0.0% (0)	7.7% (2)	8.6% (3)	1.0
Acute renal insufficiency	45.7% (21)	20.0% (1)	25.0% (1)	19.2% (5)	20.0% (7)	0.02
Chronic renal insufficiency	0.0% (0)	20.0% (1)	0.0% (0)	7.7% (2)	8.6% (3)	0.08
Abdominal aortic aneurysm	21.7% (10)	0.0% (0)	0.0% (0)	7.7% (2)	5.7% (2)	0.06
Abdominal trauma	2.2% (1)	0.0% (0)	0.0% (0)	3.9% (1)	2.9% (1)	1.0
Malignancy	15.2% (7)	20.0% (1)	25.0% (1)	34.6% (9)	31.4% (11)	0.11
Ischemic bowel	26.1% (12)	20.0% (1)	25.0% (1)	11.5% (3)	14.3% (5)	0.27
Steroid treatment	10.9% (5)	20.0% (1)	0.0% (0)	7.7% (2)	8.6% (3)	1.0
Other	17.4% (8)	40.0% (2)	0.0% (0)	11.5% (3)	14.3% (5)	0.77

**Table 4:** Outcome.

	Initially no fistula (*n* = 55)			Preexisting fistula (*n* = 26)	Any fistula overall (*n* = 35)	*P* value (any fistula versus no fistula)
	No fistula overall (*n* = 46)	Fistula during NPWT (*n* = 5)	Fistula after NPWT (*n* = 4)	Fistula before NPWT (*n* = 26)		
Abdominal closure within 30 days	84.8% (39)	40.0% (2)	100.0% (4)	76.9% (20)	74.3% (26)	0.27
Mortality	28.6% (13)	40.0% (2)	25.0% (1)	34.6% (9)	34.3% (12)	0.63

**Table 5:** Adverse events.

	Initially no fistula (*n* = 55)			Preexisting fistula (*n* = 26)	Any fistula overall (*n* = 35)	*P* value (any fistula versus no fistula)
	No fistula overall (*n* = 46)	Fistula during NPWT (*n* = 5)	Fistula after NPWT (*n* = 4)			
Death	28.6% (13)	40.0% (2)	25.0% (1)	34.6% (9)	34.3% (12)	0.63
Bleeding	15.2% (7)	0.0% (0)	25.0% (1)	3.9% (1)	5.7% (2)	0.29
Major organ failure	26.1% (12)	20.0% (1)	25.0% (1)	38.5% (10)	34.3% (12)	0.47
Acute respiratory distress	4.4% (2)	0.0% (0)	0.0% (0)	3.9% (1)	2.9% (1)	1.0
Acute respiratory failure	4.4% (2)	0.0% (0)	0.0% (0)	3.9% (1)	2.9% (1)	1.0
Cholecystitis	2.2% (1)	0.0% (0)	0.0% (0)	3.9% (1)	2.9% (1)	1.0
Mesh infection	4.4% (2)	0.0% (0)	0.0% (0)	3.9% (1)	2.9% (1)	1.0
ventral hernia	2.2% (1)	0.0% (0)	25.0% (1)	3.9% (1)	5.7% (2)	0.58
Wound infection	2.2% (1)	0.0% (0)	0.0% (0)	7.7% (2)	5.7% (2)	0.58
Candida sepsis	4.4% (2)	0.0% (0)	0.0% (0)	0.0% (0)	0.0% (0)	0.50
Sepsis	6.5% (3)	0.0% (0)	25.0% (1)	11.5% (3)	11.4% (4)	0.46
Wound healing Disorder	2.2% (1)	0.0% (0)	0.0% (0)	7.7% (2)	5.7% (2)	0.58
Other	30.4% (14)	0.0% (0)	75.0% (3)	26.9% (7)	28.6% (10)	1.0
